# Comparative Survival and Economic Benefits of Deceased Donor Kidney Transplantation and Dialysis in People with Varying Ages and Co-Morbidities

**DOI:** 10.1371/journal.pone.0029591

**Published:** 2012-01-18

**Authors:** Germaine Wong, Kirsten Howard, Jeremy R. Chapman, Steven Chadban, Nicholas Cross, Allison Tong, Angela C. Webster, Jonathan C. Craig

**Affiliations:** 1 Centre for Kidney Research, Children's Hospital at Westmead, Westmead, Australia; 2 School of Public Health, University of Sydney, Sydney, Australia; 3 Centre for Transplant and Renal Research, Westmead Hospital, Sydney, Australia; 4 Central Clinical School, University of Sydney, Sydney, Australia; 5 Department of Nephrology, Christchurch Hospital, Christchurch, New Zealand; Erasmus University Rotterdam, The Netherlands

## Abstract

**Background:**

Deceased donor kidneys for transplantation are in most countries allocated preferentially to recipients who have limited co-morbidities. Little is known about the incremental health and economic gain from transplanting those with co-morbidities compared to remaining on dialysis. The aim of our study is to estimate the average and incremental survival benefits and health care costs of listing and transplantation compared to dialysis among individuals with varying co-morbidities.

**Methods:**

A probabilistic Markov model was constructed, using current outcomes for patients with defined co-morbidities treated with either dialysis or transplantation, to compare the health and economic benefits of listing and transplantation with dialysis.

**Findings:**

Using the current waiting time for deceased donor transplantation, transplanting a potential recipient, with or without co-morbidities achieves survival gains of between 6 months and more than three life years compared to remaining on dialysis, with an average incremental cost-effectiveness ratio (ICER) of less than $50,000/LYS, even among those with advanced age. Age at listing and the waiting time for transplantation are the most influential variables within the model. If there were an unlimited supply of organs and no waiting time, transplanting the younger and healthier individuals saves the most number of life years and is cost-saving, whereas transplanting the middle-age to older patients still achieves substantial incremental gains in life expectancy compared to being on dialysis.

**Conclusions:**

Our modelled analyses suggest transplanting the younger and healthier individuals with end-stage kidney disease maximises survival gains and saves money. Listing and transplanting those with considerable co-morbidities is also cost-effective and achieves substantial survival gains compared with the dialysis alternative. Preferentially excluding the older and sicker individuals cannot be justified on utilitarian grounds.

## Introduction

End-stage kidney disease (ESKD) is a global health problem, with currently over one million people worldwide living on some form of renal replacement therapy [1]. Kidney transplantation is the treatment of choice for most patients with ESKD because of improved duration and quality of life compared with dialysis, however demand for kidneys exceeds supply in all parts of the world [2]–[4]. Despite the concerted international effort by transplant authorities to increase the number of living donor kidneys, through introduction of the paired kidney exchange and ABO incompatible programs [5]–[9], many suitable potential recipients are unable to find a suitable live donor. Deceased donor transplantation is the only other alternative for people on dialysis, but the availability of deceased donor organs is limited, with a very small proportion of the prevalent dialysis population (less than 30%, 25% and 10% in the United States, Europe and Australia, respectively) receiving a deceased donor organ each year [2]–[4].

Being on the deceased donor waiting list is a necessary step to receiving a deceased donor transplant, but the listing or de-listing criteria vary between countries and between transplant units [10]. Patient selection for listing is clinician and centre dependent, which may lead to apparent disparity in decision-making according to co-morbid status, age and socio-economic status. For example, compared to patients without diabetes, the likelihood of a diabetic being listed on the active waiting list is reduced by at least 2-fold, perhaps or fear of poor outcomes after transplantation [11]. Fewer than 5% of those on the transplant waiting list in Australia are greater than 65 years old [2]–[4], [12]. Minority groups such as Indigenous populations have fewer referrals to transplant centres, fewer complete transplant assessment and a much smaller proportion become candidates for transplantation compared to non-Indigenous populations [12]–[14]. Gender bias also exists in many countries, with recent studies reporting a consistent and significant negative association between being female and being active on the transplant waiting list [15]–[18].

Although policy-makers and transplant authorities have attempted to incorporate the principle of equity during the process of rationing scarce organ resources, such as integrating the recipients' waiting time on dialysis and recipients' immune sensitisation status in allocation algorithms [2], [3], [19], allocation policies have generally sought to maximise absolute graft and recipient survival by limiting the high-risk groups (such as diabetic patients or those with known cardiac disease), to transplantation [20]–[23]. It is these same groups of patients who have the bleakest prognosis on dialysis and may thereby achieve the greatest incremental gains from transplantation. The potential gains in life expectancy and health expenditure achieved by wait-listing people with co-morbidities compared to non-waitlisting are largely unknown. In this study, we aim to estimate the average and incremental survival benefits and health care costs of being listed on the deceased kidney donor waiting list compared to non waitlisting among individuals on dialysis, to allow better and informed decision-making around patient selection for listing.

## Methods

From a third-party payer perspective, a probabilistic model was developed to simulate the natural history of a hypothetical group of potential candidates (n = 10,000), stratified according to their underlying co-morbid states: history of cardiovascular disease, diabetes mellitus, cerebrovascular disease, obesity, current smoking and varying ages at listing and transplantation. These variables were chosen because of the reduced patient and graft survival associated with these co-morbidities [24].

### Structure of the model

**Figure 1:**
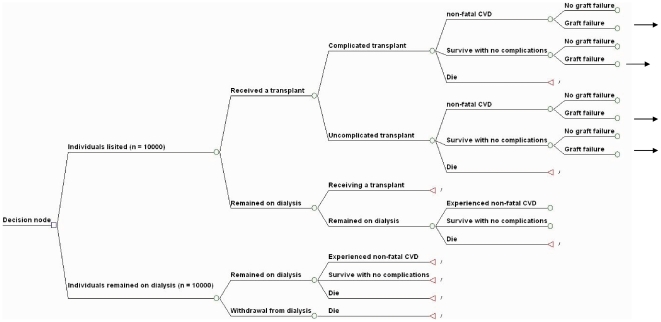
Simplified structure of the model.

The simplified structure of the model is outlined in figure 1. The cost-effectiveness model was constructed with two arms to compare the health benefits (in life-years gains) and costs of listing and transplanting potential candidates (with and without co-morbidities) with the health benefits and costs if they were to remain on dialysis. The progression of each individual through the model was depended on the age-specific transition probabilities through mutually exclusive health states of kidney transplantation and dialysis. The entire lifetime of an individual was modelled, whereby each transplant recipient was at risk of allograft failure and subsequent return to dialysis at the end of each annual cycle. The models assumed all transplant recipients were transplanted only once: all failed transplant recipients were subsequently managed on dialysis until death, and are represented by the black arrows in Figure 1. The model terminated when all potential recipients were deceased. We had set *a priori* the current Australian rate of deceased donor transplantation and did not account for the effects of variations in living donor transplantation.

We assumed the current average age of transplant (age = 45) [2]–[4], [12], the current waiting time and the annual probability of receiving a deceased donor kidney on the transplant waiting list in Australia. The risk of non-fatal cardiovascular events, and all-cause mortality, and for transplant recipients, the risk of post-transplant complications and events such as delayed graft function and wound infections, were dependent upon their underlying co-morbid health states. The model assumed a small proportion of patients with ESKD chose not to proceed with any form of renal replacement therapy. It also assumed a proportion of patients on dialysis would withdraw from dialysis each year (and opt for palliative and conservative management) and die during the concurrent year. Among those who remained on dialysis, we assumed an exponential relationship between the risk of dying from cardiac and non-cardiac causes, and the total time spent on dialysis.

### Sensitivity analysis

Assumptions were tested over a range of plausible values to assess the robustness of the uncertainties in the model's parameter estimates using sensitivity analyses. Using one-way sensitivity analyses, we identified all the influential variables within the model. Probabilistic sensitivity analysis was also undertaken. Instead of just using point estimates for parameter values, this approach assigns a distribution to each model parameter, and samples from that distribution using Monte Carlo simulation [25], [26] to estimate the expected value of each option. We used the log-normal distributions for relative risks and gamma distributions for costs, and randomly sampled over 10,000 iterations for each variable of interest. Scenario analyses were also conducted to assess the overall costs and benefits of deceased donor organ transplantation compared to being on dialysis if there were unlimited supply of deceased donor organs (i.e. no waiting time) for all individuals with varying co-morbidities.

### Ethics

Ethics approval for this study was not required as no new participants were recruited for this study. Clinical parameter estimates for the model were sourced from published literature and from de-identified data from existing data registry.

### Input parameter estimates for the model

Clinical data: A comprehensive literature search was conducted to identify the best available data on the clinical events that occurred before and after transplantation for recipients of kidney transplants and patients on dialysis. Age-specific probabilities for the following variables for transplant and dialysis patients were sourced from de-identified data from the Australian and New Zealand Dialysis and Transplant registry (ANZDATA) [27] and the National Organ Matching Service (NOMS): probability of receiving a deceased donor kidney transplant, graft failure and return to dialysis, experiencing a non-fatal cardiac event, and all-cause mortality. Other relevant data such as the probability of experiencing a transplant-associated complication including delayed graft function, acute rejection, re-hospitalisation or death was sourced from published literature. The ANZDATA Registry holds the records of all patients on renal replacement therapy in Australia and New Zealand since 1963 [27]. It contains comprehensive information such as the incidence, prevalence and outcomes data for all patients for whom indefinite renal replacement therapy is anticipated and the data is updated regularly by surveying all renal units 6 monthly before 2004, and annually since then. Multivariate Cox proportional hazard models were conducted to assess the association between cardiovascular disease, cerebrovascular disease, diabetes mellitus, smoking status, recipient age and obesity, and all-cause mortality among the listed dialysis and transplant recipients. The adjusted hazard ratios for deaths associated with the co-existing co-morbidities in transplant recipients and listed patients on dialysis were estimated using data from the ANZDATA Registry between 2004 and 2008. The adjusted hazard ratios for deaths associated with the co-existing co-morbidities in transplant recipients and patients on dialysis, and other relevant clinical data for the model are shown in Appendix S1.

Cost data: Appendix S2 shows all the cost inputs of the model. Unit costs for initial and maintenance dialysis, initial (complicated and uncomplicated), annual resources use for individuals with and without co-morbidities such as diabetes and cardiovascular disease, and maintenance costs for kidney transplantation were obtained from the Australian Refined Diagnosis Related Groups [28], the Medicare Benefits Schedule of Australia [29]–[32] and the published literature. All foreign currencies were converted to the 2008 Australian dollar using the Purchasing Power Parities [33] and the Medicare component of the Consumer Price Index (CPI).

### Model outcomes

The model outcomes included the total costs and health outcomes (expressed in life years gain) of dialysis and receiving a deceased donor kidney transplant, and the incremental costs and health benefits (in life years gains) of receiving a deceased donor kidney transplant compared to remaining on dialysis. The incremental cost-effectiveness ratio (ICER) of receiving a deceased donor kidney compared to being on dialysis was calculated for both case scenarios using the following formula:
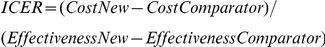
Future costs and benefits were discounted using a discount rate of 5% per annum and half-cycle corrections were employed. We used TreeAge Pro Suite 2009 (TreeAge software, Williamstown, MA, USA) [34] and Microsoft® Excel to develop and analyse the model.

## Results

Table 1 shows the total and incremental health benefits (in life years), and the total and incremental costs of waitlisting compared to non-waitlisting among individuals with ESKD and varying co-morbidities. The average gains in life years associated with listing in 45-year old potential recipient with no underlying co-morbidities compared to remaining unlisted and on dialysis were 2.41 life years, with an incremental cost-effectiveness ratio (ICER) of less than $15,000/LYS.

**Table 1:** Incremental costs and health benefits associated with listing compared to non-waitlisting among individuals with varying co-morbidities.

Characteristics of the potential recipients	Strategies	Total health benefits (LYS)	Total healthcare costs ($)	Incremental benefits (LYG)	Incremental costs ($)	ICER ($/LYS)
A 25-year old without co-morbidities	Listing	13.12	590,551	3.84	−16,272	-
	Not listing	9.28	606,823			
A 45-year old without co-morbidities	Listing	9.57	504,908	2.41	28,269	11,730
	Not listing	7.16	476,639			
A 45-year old with cardiovascular disease	Listing	7.59	479,363	1.93	27,783	14,395
	Not listing	5.66	451,580			
An obese 45 year old	Listing	8.65	556,462.	1.57	23,282	14,829
	Not listing	6.75	533,180			
A 45-year old with diabetes mellitus	Listing	6.01	360,172	1.48	13,268	8,965
	Not listing	4.52	346,904			
A 45-year old who had a stroke	Listing	8.42	539,750	1.92	24,340	12,677
	Not listing	6.50	515,410			
A 45-year old current smoker	Listing	8.81	573,295	1.81	19,690	10,878
	Not listing	7.00	553,605			
A 60-year old without co-morbidities	Listing	7.78	509,423	1.38	49,667	35,902
	Not listing	6.39	495,394			
A 60-year old with cardiovascular disease	Listing	5.86	430,074	0.88	30,350	34,489
	Not listing	4.98	399,724			
An obese 60-year old	Listing	6.67	490,699	0.62	10,954	17,668
	Not listing	6.05	479,745			
A 60-year old with diabetes mellitus	Listing	4.55	336,340	0.50	10,753	21,506
	Not listing	4.05	312,852			
A 60-year old who had a stroke	Listing	6.74	497,109	0.95	35,264	37,120
	Not listing	5.79	461,845			
A 60-year old smoker	Listing	7.26	539,394	0.96	39,278	40,915
	Not listing	6.30	500,116			

Among those with underlying co-morbidities, the incremental benefits of being listed varied between 0.50 life years in a 60-year old with diabetes mellitus to 1.93 life years in a 45-year old with cardiovascular disease. Compared to non-listing, listing an average 45-year old individual with ESKD, with and without co-morbidities on the transplant waiting list is cost-effective, and the ICERs are substantially below the accepted cost-effectiveness threshold of $50,000 per life year saved (LYS) [35].

**Figure 2:**
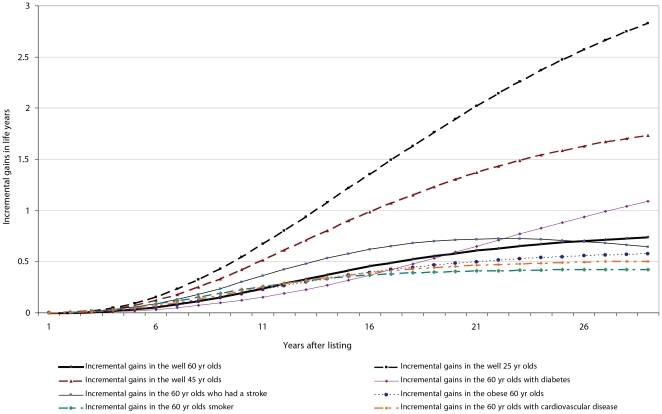
The cumulative incremental benefits of listing compared with non-waitlisting among individuals with ESKD and varying age and co-morbidities.

The cumulative incremental gains in life years from listing and transplanting individuals with varying co-morbidities are shown in Figure 2. Assuming the current waiting time on the decreased donor list, the benefits of transplantation are not evident until 4–5 years after waitlisting. A 25-year old with no co-morbidities, continues to gain survival benefits from being listed on the deceased donor list over time. This compares with listing an older recipient who is a diabetic, has a history of stroke or is a current smoker who achieve modest gains in life years (plateaus to a maximum of 0.7 to one life year gain) compared to non-waitlisting.

### Sensitivity analysis

#### Scenario analysis

**Figure 3:**
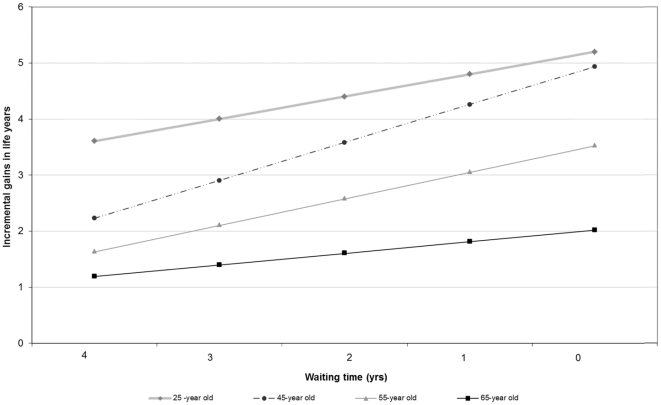
The effects of age and waiting time on the incremental benefits of listing compared with non-waitlisted individuals with ESKD.

Age at the time of listing and the waiting-time on the deceased donor transplant list are the most influential variables within the model. The extent of the variability associated with the age of listing and the waiting time on dialysis on the incremental health outcomes of listing compared with non-listing is shown in Figure 3. Assuming the median time to deceased donor kidney transplant is approximately 4–5 years in Australia, the incremental benefits of listing a 25-year old with no co-morbidities compared to non-waitlisting are 3.84 life years, with savings of over $16,000.The incremental health outcomes substantially decrease to less than 1.5 life years, with incremental costs of over $80,000 in a 65-yr old. If the waiting time of transplantation were to decrease, the maximal gains in life expectancy from transplantation compared to maintenance dialysis in a 25-year old with no co-morbidities are over 5 life years, with savings over $60,000.

Compared with maintenance dialysis, the incremental benefits associated with transplanting a 65-year old with no co-morbidities are gains of two extra life years, but with savings over $100,000. The greatest change in the incremental gains in life expectancy occurred in the “middle-age” population, varying between gains of 2 extra life years under the current waiting-time and availability of deceased organs, to over 5 extra life years if there were perfect supply of resources and no waiting time for decreased donor kidneys.

#### Probabilistic sensitivity analyses

**Figure 4:**
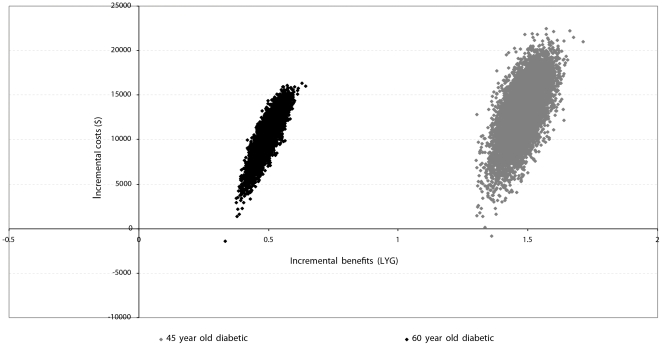
Probabilistic sensitivity analysis showing the uncertainties of the incremental costs and benefits comparing listing and non-waitlisting individuals with ESKD and diabetes.

The scatter plots shown in Figure 4 illustrate the mean incremental costs and health outcomes, and the uncertainties surrounding the mean parameter estimates associated with listing a 45- and a 60-year old potential recipient with diabetes compared to no listing. The x-axis represents the incremental gains in life years, and the y-axis represents the incremental costs of listing compared with non-waitlisting [25]. The two scattered plots are located on the northeast (NE) quadrant of the cost-effectiveness plane, with positive incremental costs and effects, indicating that listing and transplantation is both more effective but more costly than dialysis in a 45- and a 60-year old with diabetes mellitus. Compared to non-waitlisting, listing a 45-year old diabetic achieves on average, a gain of 1.5 (±0.5) extra life years compared to non-waitlisting. Listing an older person with diabetes achieves a gain of 0.62 life years, but varies between 0.45 to 0.80 life years compared with non-waitlisting.

## Discussion

Decision analytical modelling based upon current Australian outcomes of dialysis and transplantation, waitlisting and transplanting patients with ESRD, even in the presence of identified co-morbidities, is cost-effective and can be expected to achieve substantial gains of between 0.5 to more than three extra life years compared to non-waitlisting and maintenance dialysis. Given the current waiting time for decreased donor transplantation, the incremental benefits of transplantation compared to being on dialysis are not apparent until more than 4–5 years after listing. The extent of the survival benefits saved with waitlisting and transplantation is dependent on the underlying characteristics of the potential recipient, with the young and healthy achieving the greatest number of absolute and incremental gains in life years. However, the presence of the co-morbidities does not negate the benefits of waitlisting and transplantation, and indeed among those of older age with known cardiovascular disease and diabetes who are deemed suitable, listing and transplantation achieves comparable survival gains as seen in an older individual with no co-morbidities.

Previous studies have identified factors such as aging, gender, racial and socio-economic issues as being associated with the inequities in access to kidney transplantation. Once accepted on the waiting list, transplantation will achieve a gain in life expectancy of 3–15 years compared with maintenance dialysis [36]–[39], gains being dependent on donor and recipient characteristics including age at transplantation [36]–[38], [40]–[43]. Previous studies, however, have not quantified the influence of the underlying co-morbidities on the listing benefits and healthcare expenditure compared to non-waitlisting. Using decision analytic modelling, we have estimated the absolute and incremental gains in survival benefits and costs between listing compared to non-waitlisting among individuals with varying co-morbidities such as cardiovascular disease, obesity, cerebrovascular disease, smoking and aging.

Listing and transplanting the young and healthy individuals will accrue the greatest number of life years over time and achieve the greatest incremental gains in life expectancy compared to remaining on dialysis. Younger, healthier patients are often considered as “ideal” recipients who will maximally utilise the donated organs in the context of limited resources and will most likely achieve transplant success (i.e. better short and longer-term patient and graft survival compared to the older and sicker population). Whilst the absolute gains in survival among older recipients and those with co-morbidities may never be comparable to those observed for “ideal” candidates, we have shown that even in the face of the prolonged waiting-time under the current allocation algorithm, placing older individuals with co-morbidities such as cardiovascular disease and diabetes mellitus on the waiting list still achieves modest gains in survival.

Listing and transplantation affects the overall organ utilisation and therefore has an impact on all individuals with ESRD regardless of age and co-morbidities. Although the criteria to list are predominantly age and co-morbidity dependent, the allocation process is complex; and is largely dependent upon the notion of fairness and equity, where priority is largely determined by the duration of waiting time. Given the extent of the imbalance between organ demand and supply, only a small proportion of the waitlisted population will live long enough to receive a deceased donor organ before dying on the waiting list. Age and the waiting time on the deceased donor list are not unexpectedly, the most important factors that influence survival benefits from transplantation compared to being on dialysis. Under the optimistic scenario of unlimited organ supplies and no waiting-time for transplantation, transplanting the “middle to older-age” population achieves substantial relative gains in life expectancy by at least an extra 3.5 life years, compared to being on dialysis. This somewhat counterintuitive outcome is attributable to the better relative survival on dialysis among the younger population and the synergistically poorer survival outcomes among those who are older and with co-morbidities on dialysis. The annual mortality rates of older people on the waitlisting are phenomenal, varying between 5–10%, with the risk of death increasing exponentially over time, and dependent predominately on age and co-morbidities [2]–[4]. Our study findings support listing and transplanting this vulnerable group of patients early who are potentially at the greatest risk of death from cardiovascular events on dialysis. It is inevitable and foreseeable that older patients are more likely to die with a functioning graft than their younger counterparts, and many would argue that the opportunity costs (i.e. the extra life years gained from transplanting a younger person with the same deceased organ) do not support the allocation of “better” quality kidneys to older patients. Recent advocates and initiatives have suggested a change in the allocation policy to “age-match” the deceased donor organs to maximise total graft life years on a societal level [44]. Whilst the “age-match” debate is an interesting and relevant issue, this discussion is beyond the scope of our analysis.

Previous studies have shown that transplantation is good value for money, and sometimes cost-saving [45]–[47]. Conversely, dialysis is expensive, with an annual expenditure of over US$20 billion in the United States and A$ 700 million in Australia annually and the demand for renal replacement therapy worldwide is increasing. Contrary to findings from other economic models, our data have suggested transplanting older individuals with co-morbidities may not necessarily be more costly than transplanting those without co-existent illnesses, driven predominantly by the higher costs of dialysis in elderly with co-morbidities compared to those without co-morbidities [48]. Transplanting individuals with co-morbidities, such as diabetes requires additional resources, for example in management of diabetic-related diseases such as retinopathy and peripheral neuropathy, but the total cumulative costs of transplanting a diabetic are less than a non-diabetic because they die more quickly and thus mitigated the extra costs.

We would hope that the findings of our study will allow greater consistency and equity for patient entry to the waiting list and allocation of scarce transplant resources. Preferential allocation and prioritisation of scarce organs to a specific population, in particular the sicker and older population, would be controversial and would raise ethical concerns by certain groups and authorities, and does not reflect current clinical practice. Kidney transplantation is a valuable medical procedure and should be offered to those who require it equally and fairly. Given the current limited supply of and on-going excess demand for organs, the distribution process should be free from biases such as gender, age, ethnicity, income, co-morbidities and socioeconomic status.

However, historical and registry data have shown that less than 5% of those aged 65 and older are on the deceased donor waiting list and less than 1% of the older population receive a deceased donor kidney annually [2], [49], [50]. Many would argue against allowing the younger and healthier population to wait for “more years” on the transplant lists because of the cumulative “uraemic” effects on potential vascular and metabolic events, and that the greatest benefits for these younger individuals, the community, the transplant authorities, and the policy-makers are to transplant them early, to ensure maximal absolute gains in graft and patient survival. The prestige and the perceived medical excellence commonly associated with most transplant programs have prompted transplant authorities/clinicians to achieve optimal transplant outcomes by “cherry-picking” the best candidates for transplantation and not considering the overall benefits for the entire ESKD population. Increasing the numbers of patients waitlisted for transplantation through expanding the criteria to include the sicker and older individuals would likely prolong waiting times, which may adversely effect average outcomes, shorten the overall graft and patient survival, but will potentially extend the survival benefits to the disadvantaged, the sickest and the most needy ESKD population.

There are a number of limitations in our study. First, our estimates of survival gains and cost-savings are limited to patients for whom transplant clinicians had chosen to list and then transplant. For example, of all patients on renal replacement therapy who are listed as having coronary artery disease on the ANZDATA registry, it is probable that those who were transplanted on average have lesser degrees of vascular disease than those who were not listed for transplantation. Therefore, the outcome probabilities used in our models, which are based on actual outcomes, may over-estimate benefits for those with more severe disease. Second, we have not taken into consideration the benefits of living donor transplantation among those who had been listed and waiting for deceased donor transplants, and the harms, benefits and costs of multiple transplantations. Third, we have not valued outcomes in quality-adjusted life years, which may provide a more accurate assessment of both survival and quality of life outcomes in transplant recipients. There is, uniformly, a lack of utility-based quality of life data among recipients with co-morbidities. The extent of the quality of life improvement and the relative gains in quality adjusted life years post transplant in the elderly and those with co-morbid illnesses may be greater than those without co-existent diseases, rendering transplantation a more attractive option for those with co-morbidities than those without. There is therefore a need for future research to assess the utility-based quality of life of having co-morbidities such as diabetes and cardiovascular disease, in the dialysis and transplant populations to ensure a more realistic evaluation of the true impact of the survival and quality of life of having two or more chronic illnesses. Fourth, we have not considered donor factors, which may have a significant impact on the graft survival and potential survival benefits from receiving a transplant. In addition, data about the smoking status, body weight, and stroke history within the ANZDATA registry are incomplete, only collected at the start of the renal replacement therapy (and therefore may not be representative of co-morbidity status at the time of transplantation), and may be subjected to reporting bias. We have also not allowed co-morbidities to co-exist in the modelling and have not modelled re-transplantation which may potentially affect the overall survival benefits through transplantation in the ESKD population. Finally, patients' preferences and perspectives were not considered in this analysis. Previous studies have reported inconsistencies in the preferences concerning the allocation policy of transplant organs between the community, patients and healthcare professionals [50]. A recent systematic review of community preferences for organ allocation found that in addition to maximising efficiency, community preferences were also underpinned by principles relating to social valuation, moral deservingness, fair innings, and medical urgency [51].

While it is important to recognise and understand an individual's need and interests, it is also imperative to consider the interests of the wider community, particularly in the context of the limited organ supply and on-going demand. A better understanding of the absolute and incremental gains in survival and costs will help inform clinicians, decision and policy-makers about the optimal allocation of scarce organs to achieve maximal health gains, both from a societal and an individual's perspective. Excluding older and sicker patients from transplantation may disadvantage the group who actually have the greatest incremental gains in life years. The process of organ allocation is complex and requires careful distillation and consideration of all factors and available evidence, with the ultimate objective to balance the two competing interests of maximising efficiency and maintaining social justice in the distribution of limited resources.

## Supporting Information

Appendix S1 **Clinical inputs into the model.**
(DOCX)

Appendix S2 **Costs inputs into the model.**
(DOCX)
