# Supplementary material for: Comparative Survival and Economic Benefits of Deceased Donor Kidney Transplantation and Dialysis in People with Varying Ages and Co-Morbidities
Source: PLoS One. 2012 Jan 18;7(1):e29591. doi: 10.1371/journal.pone.0029591 (PMC3261160; doi:10.1371/journal.pone.0029591)
Supplement: Appendix S1 — Clinical inputs into the model. (DOCX) [file pone.0029591.s001.docx]

**Appendix S1. Clinical inputs into the model**

| **Clinical data** |  | **Base-case values (ranges used in the sensitivity analysis )** | **References** |
| --- | --- | --- | --- |
| Age-specific all-cause mortality among transplant recipients with no underlying co-morbidities | Ages  18-24  25-44  45-64  65-74  ≥ 75 | 0.0017  0.0093  0.0271  0.0616  0.1000 | [1,2] |
| Age-specific all-cause mortality among listed dialysis patients with no underlying co-morbidities | Ages  18-24  25-44  45-64  65-74  ≥ 75 | 0.0212  0.0233  0.0532  0.0763  0.2350 | [1,2] |
| Probability of experiencing a complicated event post transplant |  | 0.222 (0.15 – 0.35) | [3-6] |
| Probability of dialysis withdrawal |  | 0.02 (0.01- 0.05) | [1,2] |
| Age-specific probability of experiencing a non-fatal cardiac event among transplant recipients | Ages  18-24  25-44  45-64  65-74  ≥ 75 | 0.0698  0.0286  0.0461  0.0424  0.0336 | [1,3] |
| Age-specific probability of experiencing a non-fatal cardiac event among patients on dialysis | Ages  18-24  25-44  45-64  65-74  ≥ 75 | 0.005  0.010  0.080  0.073  0.075 | [1,3] |
| Age-specific probability of receiving a deceased kidney donor transplant | Ages  18-24  25-44  45-64  65-74  ≥ 75 | 0.148  0.102  0.068  0.015  0.005 | [1-3] |
| Age-specific probability of graft failure | Ages  18-24  25-44  45-64  65-74  ≥ 75 | 0.041  0.041  0.038  0.022  0.010 | [1,2] |
| Adjusted hazard ratios for all-cause mortality associated with different co-morbid states in kidney transplant recipients | Diabetes  Cerebrovascular disease  Cardiovascular disease  Smoking (current smoker  Obesity (BMI > 30) | 1.84 (1.60 – 2.10)  1.56 (1.19 – 2.06)  1.28 (1.08 – 1.52)  1.50 (1.31 – 1.71)  0.98 (0.93 – 1.13) | [1,2] |
| Adjusted hazard ratios for all-cause mortality associated with different co-morbid states among patients on dialysis | Diabetes  Cerebrovascular disease  Cardiovascular disease  Smoking (current smoker  Obesity (BMI > 30) | 1.61 (1.55 – 1.67)  1.33 (1.26 – 1.57)  1.70 (1.63 – 1.77)  1.13 (1.07 – 1.18)  1.75 (0.62 – 4.92) | [1,2] |
| Discount rate | Benefits  Costs | 0.05 (0.03 – 0.08)  0.05 (0.03 – 0.08) | [7] |

Reference List

1. Australia and New Zealand Dialysis and Transplant Registry (ANZDATA), Special data request (2009). 2005.

2. Australian and New Zealand Dialysis and Transplant Registry. The 30th Annual Report. 1-2-2007.

3. Australia and New Zealand Organ Donation Registry. ANZOD Registry Report 2008. 2010. Adelaide, South Australia, 5011.

4. Elli A, Traversi L, Ponticelli C (2000): Cardiovascular risk factors in renal transplant recipients. *International Journal of Artificial Organs* 23: 730-735.

5. Gill JS, Abichandani R, Kausz AT, Pereira BJ (2002): Mortality after kidney transplant failure: the impact of non-immunologic factors. *Kidney International* 62: 1875-1883.

6. Gill JS, Tonelli M, Johnson N, Kiberd B, Landsberg D, Pereira BJ (2005): The impact of waiting time and comorbid conditions on the survival benefit of kidney transplantation. *Kidney International* 68: 2345-2351.

7. Commonwealth Department of Health and Ageing. Guidelines for the pharcaceutical industry on the preparation of submissions to the Pharmaceutical Benefits Advisory Committee. 2006. Canberra, ACT: Commonwealth Department of Health and Ageing.

8. Australian Government Department of Health and Ageing: *Scedule of Pharmaceutical Benefits*. 10 A.D.

9. Australian Government Department of Health and Ageing. Medicare Benefits Schedule Book. 6-11-2006.

10. Australian Government of Health and Aging (AIHW). Australia's health 2006. 2006.

11. Australian Government Australia Institute of Health and Welfare. AR-DRG data cubes from 2006-2007. 2007.

12. Australian Institute of Health and Welfare. Australian Hospital Statistics ( 2003 - 2004). 2004.

13. Hogan C, Lunney J, Gabel J, Lynn J (2001): Medicare beneficiaries' costs of care in the last year of life. *Health Affairs* 20: 188-195.
