# Supplementary material for: Comparative Survival and Economic Benefits of Deceased Donor Kidney Transplantation and Dialysis in People with Varying Ages and Co-Morbidities
Source: PLoS One. 2012 Jan 18;7(1):e29591. doi: 10.1371/journal.pone.0029591 (PMC3261160; doi:10.1371/journal.pone.0029591)
Supplement: Appendix S2 — Costs inputs into the model. (DOCX) [file pone.0029591.s002.docx]

**Appendix S2. Costs input into the model**

| **Cost data** | | **Base-case values (ranges used in sensitivity analysis)**  **Per unit costs ($) in Australian dollars** | **References** |
| --- | --- | --- | --- |
| **Dialysis** | |  |  |
| Annual HD – hospital  Initial access  Annual HD – home  Initial access  Annual HD – satellite  Initial access  Annual CAPD  Annual APD  *Proportion of dialysis modality (Australia)*  Hospital HD  Satellite HD  Home HD  CAPD  APD  *Proportion of dialysis mortality* (New Zealand)  Hospital HD  Satellite HD  Home HD  CAPD  APD | | $85,987 ($76,584 - $95,004)  $10,896 ($9,050 - $13,578)  $50,045 ($48,670 - $55,789)  $9,990 ($7,561 - $12,758)  $52,650 ($46,735 - $65,896)  $9,990 ($8,321 - $14,890)  $60,838 ($54,434 - $78,901)  $86,905 ($80,010 - $90,908)  23%  46%  9%  10%  12%  30%  19%  16%  24%  11% | [1,2,8-12] |
| **Transplantation ( Initial – year 1)**  *Costs of standard immunosuppression*  Tac + Prednisone + MMF  CyA + Prednisone + MMF  Sirolimus + Prednisone + MMF  Everolimus + Prednisone + MMF  *Proportion of immunosupppressive therapy used*  Tac + Prednisone + MMF  CyA + Prednisone + MMF  Sirolimus + Prednisone +MMF  Everolimus + Prednisone +MMF  *Costs of induction therapies*  Basiliximab  Anti-thymoglobulin antibodies  *Costs of surgery – deceased donor + harvest*  *Costs of outpatient visits – (total = 32 per year)* | | $25,919 ($21,436 - $28,890)  $14,612 ($11,536 - $16,790)  $17,218 ($15,090 - $22,643)  $17,955 ($15,431 - $21,674)  0.25  0.50  0.13  0.12  $6,300 ($3,034 - $12,342)  $13,175 ($5,390 - $26,678)  $29,879  $2,137 ($1,890 - $26,780) | [1,2,8-12] |
| **Total costs of uncomplicated transplant** | | **$49,290** | [1,2,8-12] |
| **Costs of complicated transplant** | |  |  |
| **Above plus:** | |  |  |
| Anti-thymoglobulin antibodies | | $13,175 |  |
| Intermittent dialysis | | $6,500 |  |
| Biopsies | | $500 |  |
| **Total costs of complicated transplant** | | **$69,447** | [1,2,8-12] |
| **Transplantation (Subsequent years)**  Costs of standard immunosuppression  Tac + Prednisone + MMF  CyA + Prednisone + MMF  Sirolimus + Prednisone + MMF  Everolimus + Prednisone + MMF  Proportion of immunosupppressive therapy used  Tac + Prednisone + MMF  CyA + Prednisone + MMF  Sirolimus + Prednisone + MMF  Everolimus + Prednisone + MMF  Costs of outpatient visits (x4 per year) | | $25,919  $14,612  $17,218  $17,955  0.25  0.50  0.13  0.12  $268 | [1,2,8-12] |
| **Total costs of subsequent transplants** | | $18,446 |  |
| **Graft loss and return to dialysis** | | $17,663 (8,831 - $35,326) | [8,9,11,12] |
| **Steroid responsive acute rejection** | | $6,030 (3,489 – 12,114) | [8,9,11,12] |
| **Steroid-resistant acute rejection** | | $43,330 ($12,904 - $43,244) | [8,9,11,12] |
| **Costs of dying from non-CVD causes** | | $18,482 ($17,654 - $38,907) | [1,8,9,11-13] |
| **Cost of dying from CVD causes** | | $28,228 ($23,890 - $35,435) | [1,8,9,11-13] |
| **Costs of non-fatal CVD** | | $10,626 ($9,675 - $13,786) | [1,8,9,11-13] |
| **Annual maintenance costs for individuals with co-morbidities** | |  |  |
|  | **Diabetes mellitus** |  |  |
|  | Endocrinology review (4 visits per year) | 298.9 | [9] |
|  | Ophthalmology review (2 visits per year) | 149.4 | [9] |
|  | Laser photocoagulation therapy (2 visits per year ) | 1500 | [9] |
|  | Podiatry review (4 visits per year) | 244.4 | [9] |
|  | Dietician review (3 visits per year) | 183.3 | [9] |
|  | Annual average costs for other concomitant medications: (anti-hypertensives (15%), statins (15%), oral hypoglycaemics (40%), insulin (30%)) | 1501.6 | [8] |
|  | **Total costs** | 2827 |  |
|  | **Cardiovascular disease** |  |  |
|  | Cardiology review (4 visits per year) | 298.9 | [9] |
|  | Echocardiogram | 115.3 | [9] |
|  | ECG | 50.1 | [9] |
|  | Annual average costs for other concomitant medications  ( | 1875.6 |  |
|  | (anti-hypertensives: beta blockers, nitrates, calcium channel blockers (50%), statins (30%), anti-platelet agents (20%))  Cerebrovascular disease 1789.3 (8) |  |  |
|  | **Total costs** | 2339.9 |  |
|  | **Cerebrovascular disease** |  |  |
|  | Neurology review ( 4 visits per year) | 298.9 | [9] |
|  | Annual average costs for other concomitant medications  (anti-hypertensives (40%), statins (30%), anti-platelet agents (30%))  **Total costs** | 1825.6  2124.5 | [8] |
|  | **Obesity** |  |  |
|  | Dietician review (3 visits per year) | 183.3 | [9] |
|  | Exercise physiotherapist review ( 3 visits per year) | 183.3 | [9] |
|  | Annual average costs for other concomitant medications  (anti-hypertensives (50%), statins (50%), ) | 1251 | [8] |
|  | **Total costs** | 1617 |  |

Reference List

1. Australia and New Zealand Dialysis and Transplant Registry (ANZDATA), Special data request (2009). 2005.
Ref Type: Report

2. Australian and New Zealand Dialysis and Transplant Registry. The 30th Annual Report. 1-2-2007.

3. Australia and New Zealand Organ Donation Registry. ANZOD Registry Report 2008. 2010. Adelaide, South Australia, 5011.

4. Elli A, Traversi L, Ponticelli C (2000): Cardiovascular risk factors in renal transplant recipients. *International Journal of Artificial Organs* 23: 730-735.

5. Gill JS, Abichandani R, Kausz AT, Pereira BJ (2002): Mortality after kidney transplant failure: the impact of non-immunologic factors. *Kidney International* 62: 1875-1883.

6. Gill JS, Tonelli M, Johnson N, Kiberd B, Landsberg D, Pereira BJ (2005): The impact of waiting time and comorbid conditions on the survival benefit of kidney transplantation. *Kidney International* 68: 2345-2351.

7. Commonwealth Department of Health and Ageing. Guidelines for the pharcaceutical industry on the preparation of submissions to the Pharmaceutical Benefits Advisory Committee. 2006. Canberra, ACT: Commonwealth Department of Health and Ageing.

8. Australian Government Department of Health and Ageing: *Scedule of Pharmaceutical Benefits*. 10 A.D.

9. Australian Government Department of Health and Ageing. Medicare Benefits Schedule Book. 6-11-2006.

10. Australian Government of Health and Aging (AIHW). Australia's health 2006. 2006.

11. Australian Government Australia Institute of Health and Welfare. AR-DRG data cubes from 2006-2007. 2007.

12. Australian Institute of Health and Welfare. Australian Hospital Statistics ( 2003 - 2004). 2004.

13. Hogan C, Lunney J, Gabel J, Lynn J (2001): Medicare beneficiaries' costs of care in the last year of life. *Health Affairs* 20: 188-195.
